# Insight into the genomes of dominant yeast symbionts of European spruce bark beetle, *Ips typographus*

**DOI:** 10.3389/fmicb.2023.1108975

**Published:** 2023-04-03

**Authors:** Tian Cheng, Tereza Veselská, Barbora Křížková, Karel Švec, Václav Havlíček, Marc Stadler, Miroslav Kolařík

**Affiliations:** ^1^Laboratory of Fungal Genetics and Metabolism, Institute of Microbiology, Czech Academy of Sciences, Praha, Czechia; ^2^Department of Microbial Drugs, Helmholtz Centre for Infection Research, Braunschweig, Germany

**Keywords:** Scolytinae, gut microbiome, symbiosis, spruce, plant cell wall, detoxification, yeast, nutrition

## Abstract

Spruce bark beetle *Ips typographu*s can trigger outbreaks on spruce that results in significant losses in the forest industry. It has been suggested that symbiotic microorganisms inhabiting the gut of bark beetles facilitate the colonization of plant tissues as they play a role in the detoxification of plant secondary metabolites, degrade plant cell wall and ameliorate beetle’s nutrition. In this study, we sequenced and functionally annotated the genomes of five yeasts *Kuraishia molischiana*, *Cryptococcus* sp., *Nakazawaea ambrosiae*, *Ogataea ramenticola*, and *Wickerhamomyces bisporus* isolated from the gut of *Ips typographus*. Genome analysis identified 5314, 7050, 5722, 5502, and 5784 protein coding genes from *K. molischiana*, *Cryptococcus* sp., *N. ambrosiae*, *O. ramenticola*, and *W. bisporus*, respectively. Protein-coding sequences were classified into biological processes, cellular and molecular function based on gene ontology terms enrichment. Kyoto Encyclopedia of Genes and Genomes (KEGG) annotation was used to predict gene functions. All analyzed yeast genomes contain full pathways for the synthesis of essential amino acids and vitamin B6, which have nutritional importance to beetle. Furthermore, their genomes contain diverse gene families related to the detoxification processes. The prevalent superfamilies are aldo-keto reductase, ATP-binding cassette and the major facilitator transporters. The phylogenetic relationships of detoxification-related enzymes aldo-keto reductase, and cytochrome P450 monooxygenase, and ATP-binding cassette are presented. Genome annotations also revealed presence of genes active in lignocellulose degradation. *In vitro* analyses did not confirm enzymatic endolytic degradation of lignocellulose; however, all species can utilize and pectin and produce a large spectrum of exolytic enzymes attacking cellulose, chitin, and lipids.

## 1. Introduction

The European spruce bark beetle, or Eight-toothed spruce bark beetle, *Ips typographus* (Coleoptera: Scolytinae), infecting Norway Spruce (*Picea abies*) is one of the most destructive forest pests in Europe (Biedermann et al., 2019). Bark beetles are known to vector microorganisms that assist them during plant phloem colonization (Six, 2013; Hofstetter et al., 2015). They are sources of nutrients, i.e., sterols, vitamins, essential amino acids, and other nitrogenous compounds (Ayres et al., 2000; Bentz and Six, 2006; Veselská et al., 2019; Ibarra-Juarez et al., 2020), facilitate plant tissue colonization by detoxification of tree defense compounds (e.g., Zhao et al., 2019) or necrotize healthy plant tissue by which they also can cause tree mortality (Hofstetter et al., 2015; Li et al., 2022). It is assumed that microorganisms introduced by beetles under the bark of a tree have a greater effect on the wood modifications than beetle themselves (Hýsek et al., 2021).

Bark beetles’ habitat is a hot spot of microbial diversity and this is also true for *I. typographus*. Despite long-term investigation of its fungal assemblage, novel fungal associates of this beetle are continuously being discovered (Linnakoski et al., 2016a; Jankowiak et al., 2017). Recent studies reported spatial and temporal differences in fungal assemblages (Linnakoski et al., 2016b; Netherer et al., 2021) and showed that volatile compounds influence the beetle-fungus interactions (Kandasamy et al., 2019). Traditionally the most studied are ectosymbiotic filamentous fungi as they massively grow around and inside beetle’s galleries, often causing coloration of surrounding plant tissues or having plant pathogenic potential. *Endoconidiophora polonica* and *Ophiostoma bicolor*, together with *Grosmannia penicillata* and *G. europhioides*, are well-known filamentous fungal symbionts of *I. typographus*, having ability to detoxify host tree defense system (Zhao et al., 2019; Netherer et al., 2021). Although yeasts have been recognized as constant components in the guts of bark beetles (Rivera et al., 2009; Davis, 2015) including *I. typographus* (Chakraborty et al., 2020; Veselská et al., 2023), they were mostly overlooked in majority of the previous studies, mainly because of the difficulties in their taxonomic identification and absence of phytopathogenicity.

Recent studies (Barcoto et al., 2020; Ibarra-Juarez et al., 2020) emphasize the importance of whole microbial community, called holobiont (Six, 2013), including endosymbiotic gut inhabiting yeasts and bacteria. It was shown that gut of bark beetles provides an environment for microbial inhabitation (Engel and Moran, 2013), where plant allelochemical digestion, detoxification, and nutritional exchange take place (Linser and Dinglasan, 2014; Stefanini, 2018). Thus, gut-associated fungi can facilitate those important intestinal processes (Itoh et al., 2018). Despite the functional importance of gut inhabiting fungi, factors that maintain these complex interactions are still poorly understood (Biedermann and Vega, 2020) and there are very few studies that have comprehensively investigated bark beetles’ microbial intestinal community, particularly in terms of genomics. These studies show that yeasts together with bacteria belong to the first organisms that inhabit bark beetle galleries and synergically prepare galleries for growth of filamentous fungi (Barcoto et al., 2020; Ibarra-Juarez et al., 2020).

In the prior study (Veselská et al., 2023), we described *I. typographus* gut core microbiome based on the metatranscriptomic and DNA metabarcode analysis. We found that it is largely dominated by ascomycetous yeasts (Saccharomycetales), especially by *Kuraishia molischiana*, which took 25.6% of total fungal reads, *Nakazawaea ambrosiae*, 20.7% of the reads, *Wickerhamomyces bisporus*, 16.8% of the reads and *Ogataea ramenticola*, 2% of the reads. One unidentified species of the genus *Cryptococcus* (Basidiomycota: Tremellomycetes) with 0.1% of the reads, was selected as representative of basidiomycetous yeasts, which represent another important component of intestinal yeast diversity. On NCBI database, assembled genomes of *K. molischiana* (PPKW02; 10.36 Mb, 50.7% GC) and *O. ramenticola* (PPKK02; 12.99 Mb, 32.3% GC) are available; however, they are not annotated. Genomes of the other above-mentioned yeast species have not been sequenced yet.

In the present study, we attempt to study dominant gut-associated yeasts with an insight in their genomes. Yeast species remain an untapped source of carbohydrate-active enzymes (CAZymes) since their contribution to biomass degradation has been largely overlooked (Sun and Cheng, 2002; Zhao et al., 2014; Despres et al., 2016). In the present study, we focus on the analysis of yeasts’ CAZymes targeting plant cell wall, which is comprised mainly by pectin, cellulose, hemicellulose, other polysaccharides, lignin, lipids and proteins (Glass et al., 2013). We also searched for genes related to detoxification processes as the detoxification of plant secondary metabolites is a complex process for the bark beetles and their associated symbionts. It is performed in different physiological phases involving a variety of genes and proteins. At present, it is known that in microorganisms, enzymes such as cytochrome P450 (CYP) monooxygenases, glutathione-S-transferases (GST), multicopper oxidases, carboxylesterases, flavin-containing monooxygenases (FMO), and aldo-keto reductases (AKR) participate in xenobiotics detoxifications (Sheehan et al., 2001; Barski et al., 2008; Sehlmeyer et al., 2010; Lah et al., 2013; Ramya et al., 2016). ATP-binding cassette (ABC) transporters, major facilitator superfamily (MFS), and multidrug and toxic compound extrusion (MATE) transporters are involved in drug and stress resistance (Lah et al., 2013; Eisinger et al., 2018). Finally, we were looking for pathways related to synthesis of nutrients essential for beetle development. Such information fills the existing knowledge gaps and provides a scientific basis for future studies in the functional analysis of gut associated fungi.

## 2. Materials and methods

### 2.1. Collection, isolation, and identification of yeast strains

Strains were isolated from the gut of *I. typographus* larvae collected within a study of its intestinal microbial diversity (Veselská et al., 2023). In brief, larvae were collected during September and October 2020 in the surroundings of forest area in Nižbor (Czechia, 49°59′09.9″N 13°56′47.5″E, 390 m.a.s.l.). Larvae were taken out of the galleries and surface sterilized by subsequent washing with 70% ethanol, 2% Tween 80 (Avantor, USA) and sterile distilled water. The larvae were dissected straight after collection, their guts were homogenized and plated in various dilutions (diluted with saline) on Petri dishes with YES (5 g of yeast extract, 30 g of glucose, 20 g agar, 1 l of distilled water) and antibiotics (streptomycine and chloramphenicol, 60 mg/l). Cultivation took place for 5–7 days at 24°C in dark. DNA was extracted by Nucleo Spin kit (Machery Nagel), the ITS sequence was used for determination based on the BlastN similarity search in NCBI Genbank database. Cultures were deposited in Culture Collection of Fungi (CCF, Department of Botany, Faculty of Sciences, Charles University, Prague) as *Cryptococcus* sp. CCF 6641, *Kuraishia molischiana* CCF 6642, *Nakazawaea ambrosiae* CCF 6643, *Ogataea ramenticola* CCF 6644, and *Wickerhamomyces bisporus* CCF 6645.

### 2.2. Genome sequencing, assembly, and evaluation

Genomic DNA (gDNA) of yeasts was obtained with Nucleo Spin kit (Machery Nagel) kit. DNA yield was quantified on Qubit 2.0 Fluorometer (Thermo Fisher Scientific, Waltham, MA, USA) using Qubit dsDNA BR Assay Kit and DNA quality was checked on Nanodrop (NanoDrop™ 2000 c, ThermoFisher scientific). gDNA samples were sent to Macrogen NGS sequencing service center (Amsterdam, Netherlands) for library preparation and sequencing. A TruSeq DNA PCR free (350) library was prepared and genomes were sequenced on Illumina NextSeq 500 system with 2 × 151 nt PE, resulting in a total of 38.96 Gb and roughly 6.49 Gb read bases per sample. The quality of raw data was analyzed with FASTQC v 0.11.9 (Andrews, 2010). Sequencing adapters and low-quality reads were trimmed with Trimmomatic v 0.39 (Bolger et al., 2014), k-mer = 5 was used to remove leading and trailing bases with Phred scores lower than 33, as well as reads shorter than 150 bp. *De novo* assembly was performed using SPAdes v 3.13.1 (Bankevich et al., 2012) in Python v 3.8.10 environment with –careful setting, k-mer = 121 was selected. The completeness of assemblies was evaluated with Benchmarking Universal Single-Copy Orthologs [BUSCO v5.2.1 (Seppey et al., 2019)] to search against the saccharomycetes_odb10 database for *Kuraishia molischiana*, *Nakazawaea ambrosiae*, *Ogataea ramenticola*, and *Wickerhamomyces bisporus*. The assembly of *Cryptococcus* sp. was searched against the tremellomycetes_odb10 database. The assessment of those assemblies was performed with Quast v 5.0.2 (Gurevich et al., 2013).

### 2.3. Gene prediction and genome annotation

Gene prediction was performed using AUGUSTUS v 3.1.0 (Stanke et al., 2006) with *Saccharomyces cerevisiae* S288C as reference species. Gene Ontology (GO) annotation was carried out in eggnog-mapper v 2 (Cantalapiedra et al., 2021) with an *e*-value of 10^–3^. The GO terms were distributed by R package GO.db (Carlson et al., 2019) and plotted by ggplot (Wickham, 2006). Kyoto Encyclopedia of Genes and Genomes (KEGG) annotation was carried out with BlastKOALA (Kanehisa et al., 2016). KEGG pathways were performed with KEGG mapper (Kanehisa and Sato, 2020).

### 2.4. Identification of carbohydrate-related proteins

Carbohydrate active enzymes (CAZymes) were identified in the predicted proteins of each genome with hmmsearch in HMMER 3.3.2 (Mistry et al., 2013) against the CAZy database dbCAN2 (Zhang et al., 2018)^1^ version 8. The *e*-value was set as 1e–5.

### 2.5. Annotation and phylogenetic analysis of detoxification-related enzymes

The predicted enzymes associated with the detoxification process were identified with HMMER *E*-value 1e–5 (Mistry et al., 2013) against specific domain from Pfam database (Mistry et al., 2021): cytochrome P450 monooxygenase (PF00067), aldo-keto reductases (PF00248), carboxylesterase (PF00135), flavin-containing monooxygenase (PF00743), glutathione S-transferase (PF13409, PF14497, PF02798, PF13417, PF00043, and PF13410), multicopper-containing oxidases (PF07732, PF00394, and PF07731), ATP-binding cassette (ABC) transporter (PF00664, PF00005, PF06472, and PF00004), multidrug and toxic compound extrusion (MATE) (PF01554) and major Facilitator Superfamily (MFS) (PF07690, PF16983, PF05631, PF00083, PF12832, PF06779, PF13347, and PF05977). The results were verified and filtered with BlastP (*e*-value 1e-5) using SEG, a word-size six letters and BLOSUM62 matrix.

Multiple sequence alignment was carried out with MAFFT v 7.453 (Rozewicki et al., 2019) using maxiterate 1000. The phylogenetic trees were performed with PhyML v 3.0 (Guindon et al., 2010). The best evolutionary model for protein sequences was calculated with Akaike method and likelihood-based method was selected as aLRT SH-like and bootstrap analysis repeated for 100 times. According to the BlastP results and phylogenetic analysis, enzymes of aldo-keto reductases, cytochrome P450, and ABC transporter family were classified into specific groups. Phylogenetic trees were visualized and edited with iTOL (Letunic and Bork, 2021).

### 2.6. Prediction of localization of detoxification-related enzymes

The subcellular localization of detoxification related enzymes was predicted with Protcomp-AN v 6.0 (Klee and Ellis, 2005), molecular mass isoelectric point (pI) was performed with Protein isoelectric point calculator (Kozlowski, 2016).

### 2.7. Extracellular enzyme activities

As our functional analysis predicts presence of pathways for the degradation of plant cell wall components, in the next step we verified them by *in vitro* degradation tests. The analyzed enzymes are listed in Table 1. Activity of endolytic enzymes was assessed using various chromogenic substrates (Megazyme, Bray, Ireland), whereas activity of exolytic enzymes was assessed using fluorogenic substrates (Sigma-Aldrich). For endolytic enzyme analyses, yeasts were cultivated in submerged media in 250 ml Erlenmeyer flasks on rotary shaker (200 rpm) at 24°C. Basic medium (BM) consisted of 1 g pepton, 1 g yeast extract, 1 g (NH_4_)_2_HPO_4_, 0.5 g K_2_PO_4_, 0.5 g MgSO_4_.7H_2_O and substrates specific to the enzyme tested [3 g of cellulose, 6 g xylan birchwood, 15 g galactomannan (Locust beam gum), 6 g of starch, 1.5 g of amylopectin and 13.5 g of dextran] in 1 l of McIlvaine buffer, pH 4.5. The cultivation time was determined using the glass tube test. Glass tubes containing water agar (20 g agar in 1 L distilled water) were overlaid with a thin layer of a specific chromogenic substrate dissolved in water agar and the agar surface was inoculated with the tested fungi and cultured at 24°C. Enzymatic degradation was manifested by diffusion of the chromogenic substrate into the water agar. At this point, the submerged cultivation in the Erlenmeyer flask was stopped. If no staining of the water agar was evident after 14 days of cultivation in the glass tubes, submerged cultivation was also stopped and enzyme activities were evaluated. Enzymes were extracted into extraction buffers as it is recommended by manufacturer for 2 h at 4°C. After extraction, the mixture was centrifuged for 10 min at 3,000 rpm to exclude the medium and fungal cells. The supernatant containing extracellular enzymes was further filtered through filter paper to remove remaining solid parts. We used manufacturer’s protocols for measurement and calculation of enzymatic activities. *Trichoderma viride* CCF 4516 was used as a positive control for enzymatic degradation of cellulose, xylan and casein as it is known to degrade these substrates (Gomes et al., 1992; Kredics et al., 2005). Sterile media without inoculated fungi served as blanks.

**TABLE 1:** Extracellular enzyme analyzed in our study.

Target	Enzyme (endo or exolytic)	Substrate
Cellulose	*endo*-1,4-β-D-glucanase (endo)	Azo-CM-Cellulose
	Cellobiohydrolase (exo)	MUF-β-D- cellobioside
	β-glucosidase (exo)	MUF-β-D-glucopyranoside
Hemicellulose	*endo*-1,4-β-D-xylanase (endo)	Azo-Xylan (Birchwood)
	β-xylosidase (exo)	MUF-β-D-xyloside
Pectin	*endo*-1,4-β-D-mannanase (endo)	Azo-Carob Galactomannan
	*endo*-1,5-α-L-arabinanase (endo)	Red Debranched Arabinan (Sugar Beet)
	β-galactosidase (exo)	MUF-α-D-galactoside
	β-mannosidase (exo)	MUF-β-D-mannoside
	α-glucuronidase (exo)	MUF-α-D-glucuronide hydrate
Starch	α-amylase (endo)	Red Starch
	Pullulanase (endo)	Red Pullulan
Lignin	Laccase (endo)	ABTS
Proteins	*endo*-protease (endo)	Azocasein
Chitin	Chitinase (exo)	MUF-β-D-N.N-diacetylchitobiose hydrate
Phosphate monoester	Acid phosphomonoesterase (exo)	MUF-phosphate
Triglycerides	Lipase (exo)	MUF-oleate

MUF, 4-Methylumbelliferone; ABTS, 2,2′-azino-bis(3-ethylbenzothiazoline-6-sulfonic acid).

All MUF based substrates and ABTS were purchased from Sigma-Aldrich, other substrates were purchased from Megazyme.

To measure exolytic activity, yeast strains were also cultured in submerged medium in 250 ml Erlenmeyer flasks on a rotary shaker (200 rpm) at 24°C. The medium consisted of yeast extract (5 g/l), peptone (2 g/l), glucose (2 g/l), casein (2 g/l), cellulose (3 g/l), lignin (3 g/l), starch (3 g/l), lime phloem (63 g/l). The yeast grew for 14 days. Sodium acetate, pH 4.5, was used for enzyme extraction. The fluorescence of the released reaction products was measured as previously described (Baldrian, 2009) using a method adapted from Vepsäläainen et al. (2001). The fluorescence value of each MUF fluorogenic substrate was corrected by subtracting the background fluorescence of the growth medium. Laccase activity (EC 1.10.3.2) was measured by monitoring the oxidation of ABTS [2,2-azinobis-3-ethylbenzothiazoline-6-sulfonic acid (Bourbonnais and Paice, 1990) in citrate-phosphate buffer (100 mM citrate, 200 mM phosphate, pH 5.0)]. The formation of the resulting green dye was evaluated spectrophotometrically at 420 nm.

## 3. Results

### 3.1. Genome sequencing and assembly

General genome features, e.g., genome size, scaffolds number, N50, L50, number of predicted genes, GC content, completeness of assembly and NCBI accession numbers are summarized in Table 2. The number of reads generated by Illumina sequencing ranged from 39,806,602 to 47,849,884 reads with an average count of 43,006,421. Established genomes range in size from 9.86 to 18.3 Mb. Genomes assembled with Spades resulted in 315–799 scaffolds and were predicted to contain 5,314–7,050 genes, and GC contents from 34.5 to 59.3%. The N50 of five genomes range from 197,085 to 1,183,430, while L50 align from 5 to 26. BUSCO assessment results showed that these assembles are in high quality with around 96% of genes were covered for all five species.

**TABLE 2:** Genome assembly statistics of yeasts isolated from the gut of *Ips typographus.*

Species	Assembly size (Mb)	Number of scaffolds	N50 (bp)	L50	Predicted genes	GC (%)	BUSCO (%)	NCBI WGS project
*K. molischiana* CCF 6642	9.86	354	475.982	8	5.314	54.8	98.4	JANBXH01
*Cryptococcus* sp. CCF 6641	18.30	629	235.228	26	7.050	59.3	95.9	JANBXI01
*N. ambrosiae* CCF 6643	13.20	315	546.084	5	5.722	42.3	98.6	JANBXG01
*O. ramenticola* CCF 6644	12.20	269	1.183.430	4	5.502	34.5	98.4	JANBXF01
*W. bisporus* CCF 6645	11.70	628	300.060	13	5.784	35.9	97.5	JANBXE01

### 3.2. GO enrichment and KEGG pathway annotation

**FIGURE 1:**
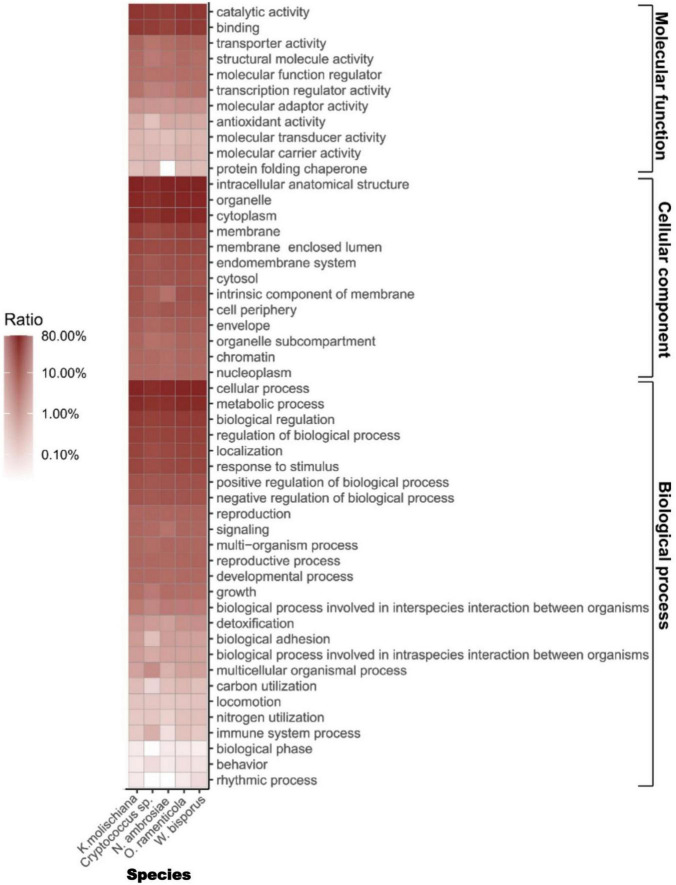
Gene Ontology (GO) terms enrichment in five genomes. The GO annotation is presented in *Y*-axis, while species names were labeled in *X*-axis.

Gene Ontology enrichment analysis indicated that about 63.06, 32.96, 59.14, 63.54, 70.76, and 70.65% genes from *K. molischiana*, *Cryptococcus* sp., *N. ambrosiae*, *O. ramenticola*, and *W. bisporus* were mapped to GO terms (Figure 1 and Supplementary Table 1). They were matched to three ontologies: molecular function, cellular component, and biological process. They were highly enriched in cellular process, metabolic process, intracellular anatomical structure, organelle, cytoplasm, binding and catalytic activity. *Cryptococcus* sp. differed from the other considered species in several functions, such as antioxidant activity, biological adhesion, multicellular organismal process, carbon utilization, immune system process, biological phase and rhythmic process.

**FIGURE 2:**
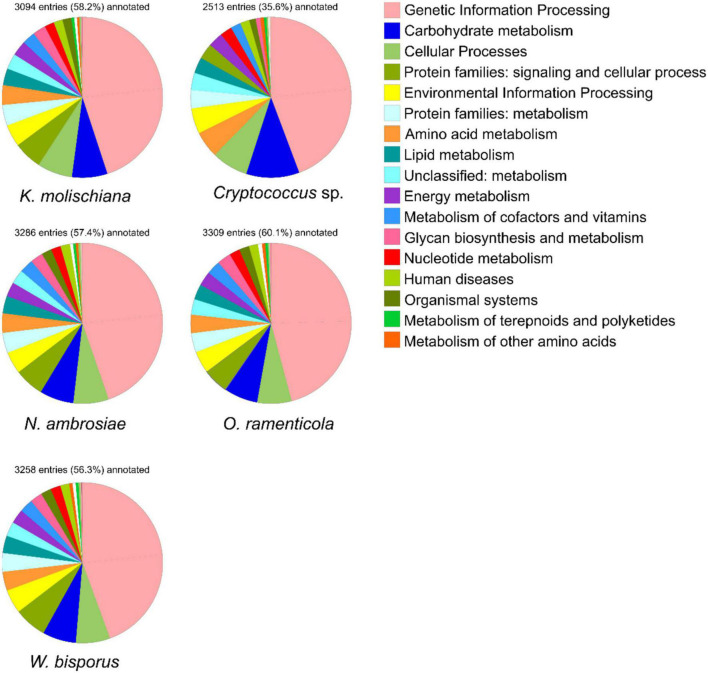
Kyoto Encyclopedia of Genes and Genomes (KEGG) pathway annotation.

To investigate the function of proteins in those five species, the annotation based on BlastKOALA (Kanehisa et al., 2016) against the KEGG database was performed. It was possible to annotate 58.2, 57.4, 60.1, 56.3, and 35.6% of conserved sequences from *K. molischiana*, *N. ambrosiae*, *O. ramenticola*, *W. bisporus*, and *Cryptococcus* sp., respectively, Figure 2. As a result, the highest enriched system was “Genetic information processing,” followed by “Carbohydrate metabolism,” and “Cellular processes.” Vitamin B6 compounds, are synthetized and metabolized in all five species (Supplementary Figure 1). Additionally, essential amino acids such as arginine, lysine, phenylalanine, tyrosine, valine, leucine, and isoleucine are biosynthesized in these yeast strains as well (Supplementary Figure 2).

### 3.3. Identification of carbohydrate-active enzyme (CAZyme)

**FIGURE 3:**
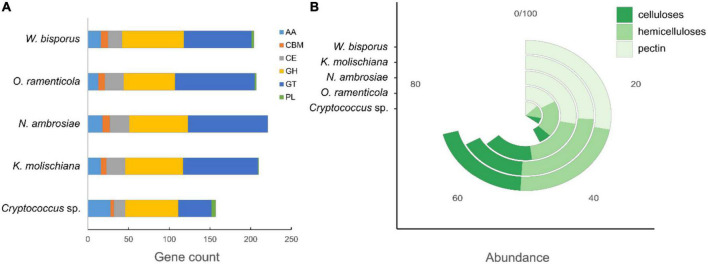
Carbohydrate active enzymes (CAZymes) present in the yeasts’ genomes. **(A)** Comparison of CAZymes classes in five species. **(B)** Comparison of gene counts related to celluloses, hemicelluloses and pectin in six species. GT, glycosyl transferase; GH, glycoside hydrolase; CE, carbohydrate esterase; CBM, carbohydrate-binding module; PL, polysaccharide lyase; AA, auxiliary activity.

The carbohydrate-active enzyme (CAZyme) gene contents were predicted for all five genomes (Figure 3A, Supplementary Figure 3 and Supplementary Table 2) in this study. These CAZymes are classified into six classes: auxiliary activity (AA), carbohydrate-binding module (CBM), carbohydrate esterase (CE), glycoside hydrolase (GH), glycosyltransferase (GT), and polysaccharide lyases (PL). *Nakazawaea ambrosiae* has more CAZymes (221) than the other four studied species, while the *Cryptococcus* sp. has the smallest CAZymes count (157). GH accounted for almost half of the identified enzymes (83–98) in those species except for *Cryptococcus* sp. (41), followed by GH (63–79), AA (13–28), CE (14–24), and CBM (4–9). PL (0–5) comprised only a few genes and is absent in *Nakazawaea ambrosiae*.

The number of identified genes regulating plant cell wall-degrading enzymes varies in five yeast species (Figure 3B). *Wickerhamomyces bisporus* has the highest (71) and *Cryptococcus* sp. has the least number of genes (35). The number of genes related to cellulose degradation is lower than those related to hemicelluloses and pectin degradation in all genomes.

### 3.4. Analysis of genes related to detoxification

More than 200 detoxification related genes were identified from each species, *K. molischiana* (225), *N. ambrosiae* (235), *O. ramenticola* (200), and *W. bisporus* (276), except for *Cryptococcus sp.* (110) (Table 3 and Supplementary Table 3).

**TABLE 3:** Number of genes involved in the detoxification process.

	*K. molischiana* CCF 6642	*Cryptococcus* sp. CCF 6641	*N. ambrosiae* CCF 6643	*O. ramenticola* CCF 6644	*W. bisporus* CCF 6645
Aldo-keto reductases	17	8	21	19	25
Carboxylesterase	6	2	6	1	3
Cytochrome P450 monooxygenase	4	6	5	4	5
Flavin-containing monooxygenase	3	5	5	3	15
Glutathione S-transferase	6	4	9	8	11
Multicopper oxidases	2	3	4	2	5
Transporter ABC	35	37	30	29	31
Multidrug and toxic compound extrusion (MATE)	5	0	5	5	6
Major facilitator Superfamily (MFS)	132	41	127	113	154

**FIGURE 4:**
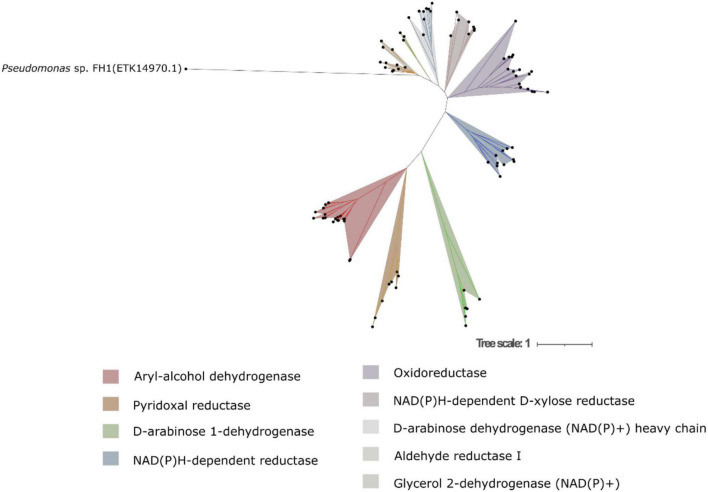
Maximum-likelihood tree of aldo-keto reductases predicted from all five yeast genomes.

We predicted presence of 115 aldo-keto reductase enzymes, with lengths from 107 to 599 amino acids, pIs from 4.62 to 9.00, and predicted molecular weights between 12.02 and 65.57 kDa. They were predicted to be localized in the cytoplasm, plasma membrane, nucleus, mitochondria, vacuoles, and the bonding membranes of the Golgi apparatus. They can be classified into 9 subfamilies (Figure 4), aryl-alcohol dehydrogenases, D-arabinose 1-dehydrogenases, oxidoreductases, and NAD(P)H-dependent D-xylose reductases. They were found in all five genomes with 22, 7, 24, and 10 members, respectively. There are 9, 14, 10, and 16 genes identified in subgroups pyridoxal reductases, NAD(P)H-dependent reductases, D-arabinose 1-dehydrogenases (NAD(P)+) heavy chain, and glycerol 2-dehydrogenases (NAD(P)+), respectively. These subgroups were absent in *Cryptococcus* sp. Three genes putatively encoding for aldehyde reductase I were found in *Cryptococcus* sp. and *N. ambrosiae.*

**FIGURE 5:**
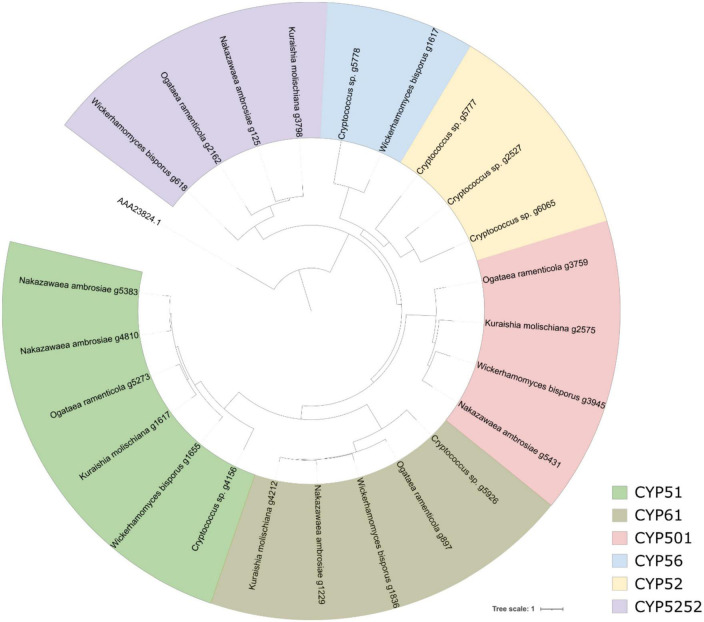
Maximum-likelihood tree of P450 predicted from all five yeast genomes.

We identified 29 cytochrome P450 (CYP) monooxygenases with amino acids lengths from 126 to 591, molecular weights from 13.78 to 66.48 kDa, and pIs from 4.9 to 8.51. They were predicted to localize in cytoplasm, plasma membrane, mitochondria, bounding membrane of mitochondria and endoplasmic reticulum. The topology tree of CYP monooxygenases showed that they can be classified into six subfamilies (Figure 5), with 7, 6, 5, 2, 4, and 5 members for CYP51, CYP61, CYP501, CYP56, CYP52, and CYP5252, respectively. Sequences in the CYP51 and CYP61 subfamilies were observed in all five species. CYP501 and CYP5252 were absent in *Cryptococcus* sp. CYP52 only existed in *Cryptococcus* sp., while CYP56 was present in *W. bisporus* genome.

**FIGURE 6:**
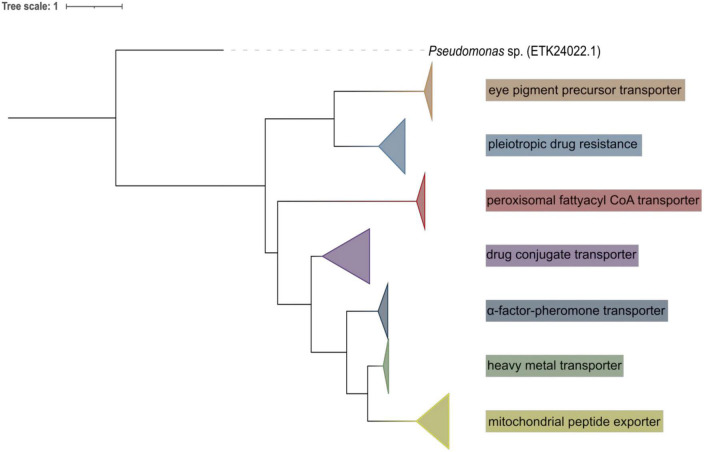
Maximum-likelihood tree of ABC transporter predicted from all five yeast genomes.

ATP binding cassette (ABC) transporters were predicted from those five genomes, they can be classified into seven subfamilies (Figure 6): 35 pleiotropic drug resistance, 10 eye pigment precursor transporter, 62 drug conjugate transporter, 7 heavy metal transporter, 13 α-factor-pheromone transporter, 43 mitochondrial peptide exporter, and 11 peroxisomal fatty acyl CoA transporter. Their amino acid lengths varied from 120 to 2058 aa, molecular weights from 13.14 to 230.256 kDa, pIs from 4.23 to 9.06. Their predicted subcellular localizations are cytoplasmic, mitochondrial, plasma and endoplasmic reticulum membranes, peroxisomal and vacuolar.

We predicted the presence of 21 carboxylesterases with 109 to 566 amino acids and molecular weight of 12.10 to 62.49 kDa. Then we predicted 46 flavin-containing monooxygenases with 137–647 amino acids, and molecular weights 15.86–72.63 kDa. Glutathione S-transferases were predicted with number of amino acids varying from 111 to 1006 and molecular weights 12.21–110.52 kDa. We also predicted 21 multicopper oxidases with 152–722 amino acids and 17.48–76.62 kDa, 38.10% of them were predicted as extracellular enzymes. The number of amino acids of multidrug and toxic compound extrusion (MATE) ranged from 361–1018 with molecular weights 39.43–113.68 kDa. Major Facilitator Superfamily (MFS), which contains 720 sequences, has 92–1863 amino acids with molecular weights 10–207.86 kDa (Supplementary Table 3).

### 3.5. Extracellular enzyme activities

We measured the activities of endo- and exolytic enzymes degrading complex components of plant tissues (see Table 4 for results) as functional analysis predicted the presence of these genes in yeast genomes. We found that the yeast species studied do not produce endolytic enzymes, with the exception of endo-1,4-β-D-mannanase, which cleaves pectin. However, all yeasts were strong producers of exolytic enzymes. *Kuraishia molischiana* was the only species producing all the enzymes tested, especially β-glucosidase, cellobiohydrolase, β-xyloside and β-mannosidase. *Nakazawaea ambrosiae* was the strongest producer of acid monophosphatase, lipase and β-mannosidase. *Wickerhamomyces bisporus* mainly secreted β-glucosidase and lipase, and *O. ramenticola* β-glucosidase and β-mannosidase. *Cryptococcus* sp. was the weakest of the yeasts in enzyme activities and produced mainly β-glucosidase and lipase. We successfully measured the enzyme activities of the positive control *Trichoderma viride*, suggesting that the absence of endolytic enzymes in the yeast species was not a methodological error.

**TABLE 4:** Enzymatic activities of the studied yeasts.

Target substrate	Enzyme (endo, or exolytic)	Enzyme activities [mU]					
		*Cryptococcus* sp. CCF 6641	*K. molischiana* CCF 6642	*N. ambrosiae* CCF 6643	*O. ramenticola* CCF 6644	*W. bisporus* CCF 6645	*T. viride* CCF 4516
Cellulose	*endo*-1,4-β-D-glucanase	0.0	0.2	0.0	0.0	0.0	5.3
	Cellobiohydrolase	0.1	239.0	44.9	1.2	0.2	326.0
	β-glucosidase	14.3	493.7	352.2	50.9	111.1	358.9
Hemicellulose	*endo*-1,4-β-D-xylanase	0.0	0.0	0.0	0.0	0.0	8.5
	β-xylosidase	2.8	504.0	11.5	8.8	7.7	148.8
Pectin	*endo*-1,4-β-D-mannanase	1.5	1.0	0.6	1.2	1.0	0.5
	*endo*-1,5-α-L-arabinanase	0.0	0.0	0.0	0.0	0.0	0.0
	β-galactosidase	0.0	2.4	17.5	0.0	1.1	306.3
	β-mannosidase	0.0	12.7	15.4	8.6	0.1	10.0
	α-glucuronidase	0.0	1.1	0.1	0.0	0.6	431.1
Starch	α-amylase	0.0	0.0	0.1	0.0	0.0	0.0
	Pullulanase	0.0	0.0	0.0	0.0	0.0	0.0
Lignin	Laccase	0.0	0.0	0.0	0.0	0.0	0.0
Proteins	*endo*-protease	0.0	0.0	0.0	0.0	0.0	5.3
Chitin	Chitinase	0.0	1.3	0.0	1.1	0.5	99.1
Phosphate monoester	Acid phosphomonoesterase	0.0	3.3	221.5	0.0	1.0	202.2
Triglycerides	Lipase	11.7	4.5	507.9	2.0	224.8	209.7

*Trichoderma viride* was used as a positive control for cellulose, hemicellulose, pectin, proteins, chitin and lipids.

## 4. Discussion

Central Europe is currently facing outbreaks of spruce bark beetles, of which *Ips typographus* is the most common and widespread species (Biedermann et al., 2019). In the previous study (Veselská et al., 2023) we found that ascomycetous yeasts, namely, *K. molischiana*, *N. ambrosiae*, *O. ramenticola*, and *W. bisporus* dominate the gut fungal microbiome of *I. typographus*. The set was supplemented by a representative of basidiomycetous yeasts, *Cryptococcus* (Tremellales). In the present study we sequenced and annotated genomes of these yeasts to infer their possible roles in the bark beetle habitat.

Bark beetles face harsh conditions when they colonize rigid plant tissues which are full of toxic secondary metabolites related to tree defense against intruders (e.g., Netherer et al., 2021). The bark beetles associated filamentous fungi facilitate colonization as these fungi are often involved in detoxification and/or cause necrosis of the plant tissues (Hofstetter et al., 2015Include “Hofstetter, 2015” in the reference list.; Zhao et al., 2019; Li et al., 2022). Carbohydrate-active enzymes (CAZymes) play a vital role in the depolymerization of the complex lignocellulosic polysaccharides (Arntzen et al., 2020; Chettri et al., 2020). The genome analysis of the five yeast species revealed the presence of diverse CAZyme-encoding genes, which constituted around 3.6% of all putative proteins identified. This proportion is similar to phytopathogenic species associated with bark beetles *Ophiostoma novo-ulmi* (Comeau et al., 2015) and *Grosmannia clavigera* (DiGuistini et al., 2011), but lower compared to other Pezizomycotina (Comeau et al., 2015). The analyzed yeasts lack endolytic enzyme cleaving lignocellulose in our *in vitro* tests, which does not allow them for efficient degradation of these polymers. On the other side, they cleave pectin and degrade carbohydrate from the terminal ends by exolytic enzymes, what allows to utilize simpler carbohydrates. These results are in concordance with previous findings on microbial enzymatic functions in bark beetles’ galleries revealed by Barcoto et al. (2020) and Ibarra-Juarez et al. (2020). These authors found that the associated bacterial community has greater potential than fungi to cleave structural components of plant tissues and that the whole holobiont forms a biofilm in which species can benefit from each other from their enzymatic capabilities.

Besides CAZymes, genes important in the colonization of plant tissues are those involved in the fight against oxidative stress or the detoxification of plant tissues and those having protective functions (DiGuistini et al., 2011; Comeau et al., 2015; Schuelke et al., 2017). Analyzed yeasts possess genes known to be involved in the degradation of toxic compounds, like aldo-keto reductases (e.g., Barski et al., 2008), glutathione S-transferase (Sheehan et al., 2001), cytochrome P450 monooxygenases (CYPs) (Lah et al., 2013), carboxylesterases (Ramya et al., 2016), and flavin-containing monooxygenase (Sehlmeyer et al., 2010), and genes for drug and stress resistance like, ABC transporter, multidrug and toxic compound extrusion and major facilitator superfamily (Lah et al., 2013; Eisinger et al., 2018). All six CYP subfamilies identified in the present study have already been described in yeast genomes (Linder, 2019). Some of them (CYP51, CYP61, and CYP56) are needed for yeast growth and development (Linder, 2019) as they are involved in the biosynthesis of ergosterol (Turi et al., 1991; Kelly et al., 1995; Chen et al., 2014), and outer layer of the yeast spore wall (Briza et al., 1994). CYP52 family is involved in alkanes and fatty acid assimilation (Sanglard and Loper, 1989). Role of the other two identified cytochromes, CYP501 and CYP5252, is unknown (Linder, 2019). A recent study (Naseer et al., 2023) shows that *I. typographus* itself has a gene repertoire related to the detoxification of plant secondary metabolites. Therefore, it awaits unraveling how the microbial and insect detoxification pathways are interconnected whether complementarily or redundantly.

Associated fungi assist beetles not only in plant tissue colonization but also in the nutrition. Plant tissues have a very low nitrogen to carbon ratio (Yang and Luo, 2011), thus larvae have to bore long tunnels to satisfy this need. Fungi colonizing beetles’ galleries and surrounding tissues enhance nitrogen budget, which increases beetles’ fitness (Ayres et al., 2000). All analyzed yeasts in the present study possess pathways for synthesis of all essential amino acids. At the same time these yeasts are able to synthesize vitamin B6, which insect also must acquire through diet (Douglas, 2017). Thus, our results show that yeasts can be important source of nutrients.

In conclusion, genome analyses of the symbiotic yeasts *K. molischiana*, *Cryptococcus* sp., *N. ambrosiae*, *O. ramenticola*, and *W. bisporus* isolated from the gut of *Ips typographus* indicate that these species can synthesize essential amino acids and vitamin B6, which are important for the development of *I. typographus*. In addition, they possess a wide range of genes associated with plant tissue detoxification, which may protect bark beetle against induced plant defense system. They also encode enzymes active on plant cell wall; however, our data suggest that yeasts don’t use lignocellulose directly for nutrition. They probably use products of lignocellulose degradation effectuated by other associated microorganisms. The genome sequences generated in our study provide a better understanding of symbiotic relationship between microorganisms in the gut of bark beetle and their host.

## Data availability statement

The datasets presented in this study can be found in online repositories. The whole-genome assemblies of *K. molischiana*, *Cryptococcus* sp., *N. ambrosiae*, *O. ramenticola*, and *W. bisporus* have been deposited on the National Center for Biotechnology Information (NCBI) under the Bioproject number PRJNA841440, BioSample numbers are SAMN28560960 for *K. molischiana*, SAMN28560962 for *O. ramenticola*, SAMN28560961 for *N. ambrosiae*, SAMN28560963 for *W. bisporus*, SAMN28560959 for *Cryptococcus* sp. NCBI WGS projects are JANBXH01 for *K. molischiana*, JANBXF01 for *O. ramenticola*, JANBXG01 for *N. ambrosiae*, JANBXE01 for *W. bisporus*, and JANBXI01 for *Cryptococcus* sp.

## Author contributions

MK and MS acquired the funding. MK and TV planned the research and revised the manuscript. BK, VH, and KŠ isolated the yeast strains from the gut of bark beetle. VH isolated the DNA from yeast strains. BK submitted for genome sequencing. VH and TV measured and analyzed enzyme activities. TC analyzed genomic data and draft the manuscript. All authors approved the manuscript.

## Footnotes

1
https://bcb.unl.edu/dbCAN2/download/Databases
