# Supplementary material for: Insight into the genomes of dominant yeast symbionts of European spruce bark beetle, Ips typographus
Source: Front Microbiol. 2023 Apr 3;14:1108975. doi: 10.3389/fmicb.2023.1108975 (PMC10106607; doi:10.3389/fmicb.2023.1108975)
Supplement: Supplementary file 1 [file Data_Sheet_1.docx]

Supplementary Material

Insight into the genomes of dominant yeast symbionts of European spruce bark beetle, *Ips typographus*

Tian Cheng^1,2^, Tereza Veselská^1^, Barbora Křížková^1^, Karel Švec^1^, Václav Havlíček^1^, Marc Stadler^2^, Miroslav Kolařík^1*^

^1^Laboratory of Fungal Genetics and Metabolism, Institute of Microbiology, Czech Academy of Sciences, Praha, Czech Republic

^2^Helmholtz Centre for Infection Research, Braunschweig, Germany

***Correspondence:**Miroslav Kolařík
mkolarik@biomed.cas.cz

**Supplementary Figure 1.** Vitamin B6 metabolism in yeast species.


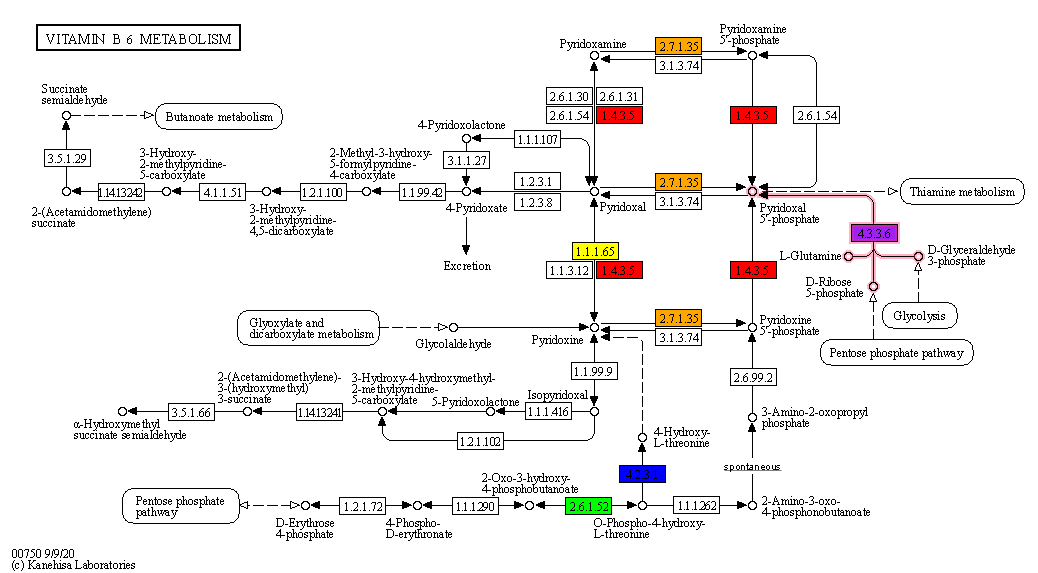


Vitamin B6 metabolism in *Nakazawaea ambrosiae* & *Ogataea ramenticola*


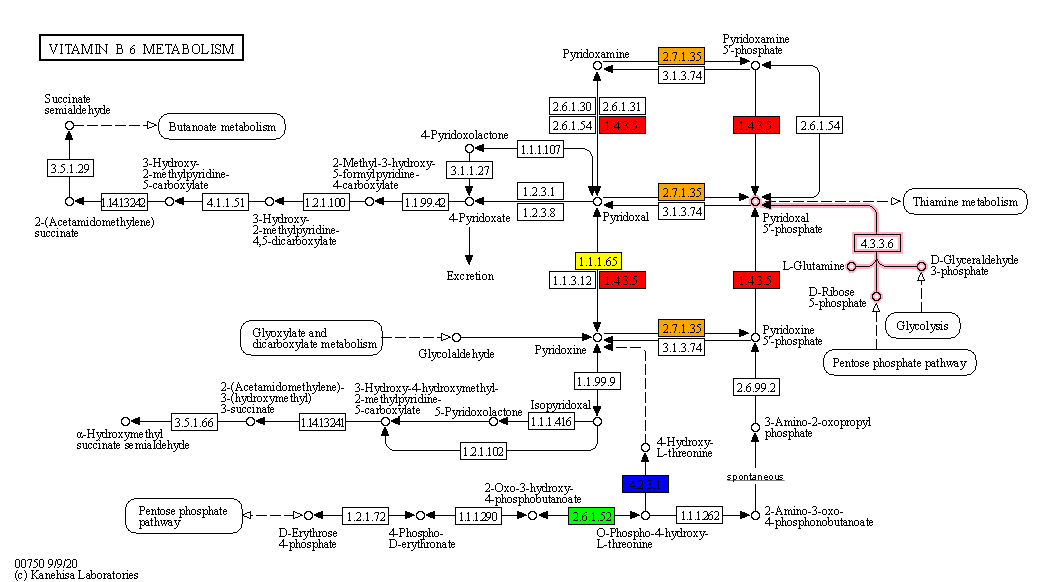


Vitamin B6 metabolism in *Kuraishia molischiana* & *Wickerhamomyces bisporus*


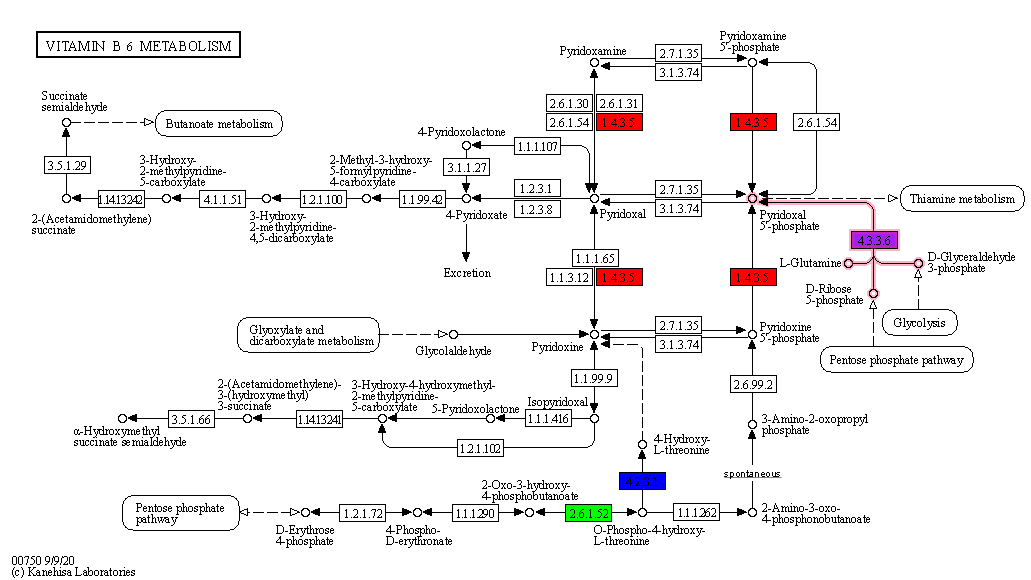


Vitamin B6 metabolism in *Cryptococcus* sp.

**Supplementary Figure 2.** Biosynthesis of amino acids. Green boxes imply the genes present in all sequenced yeast species


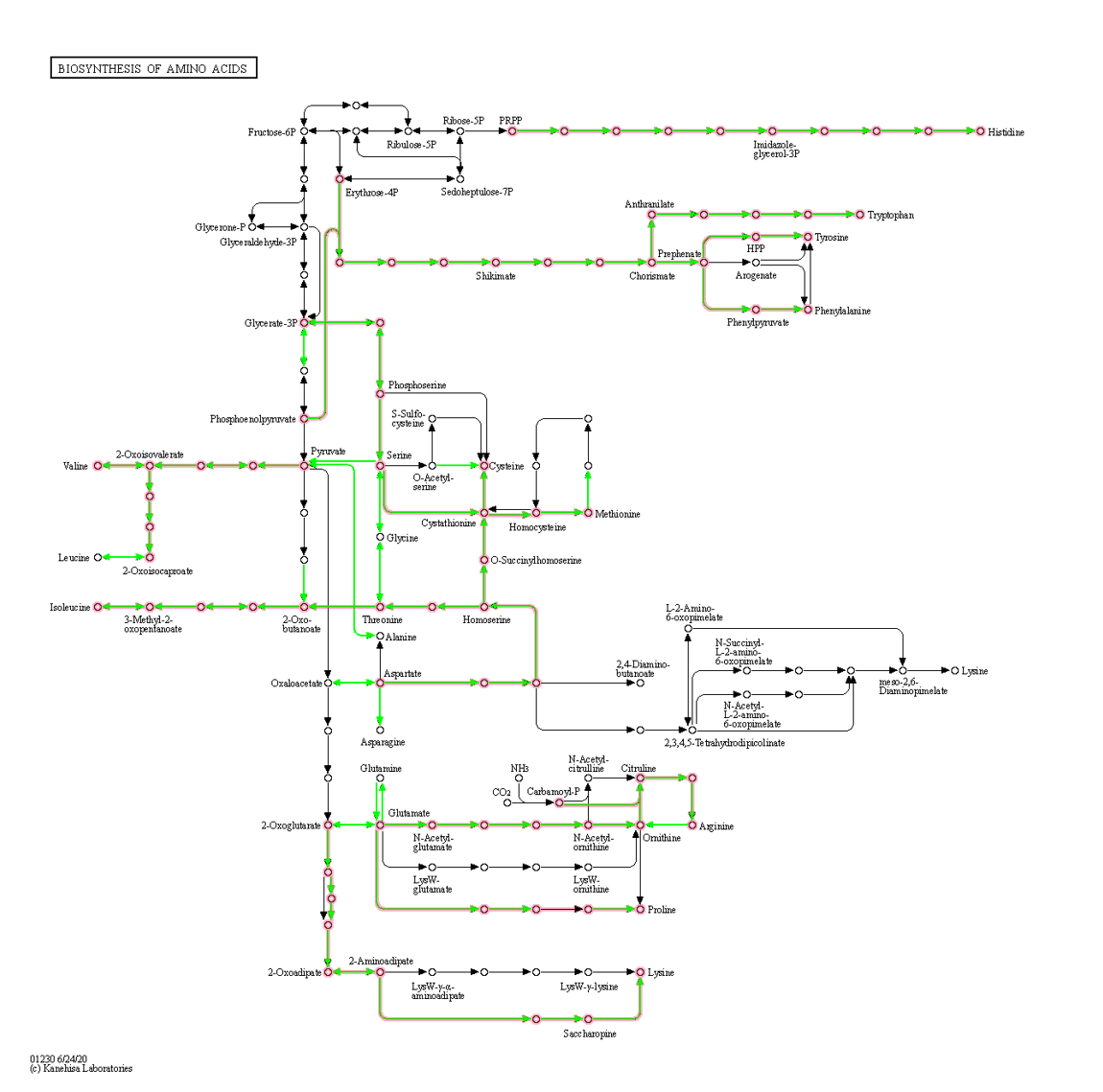


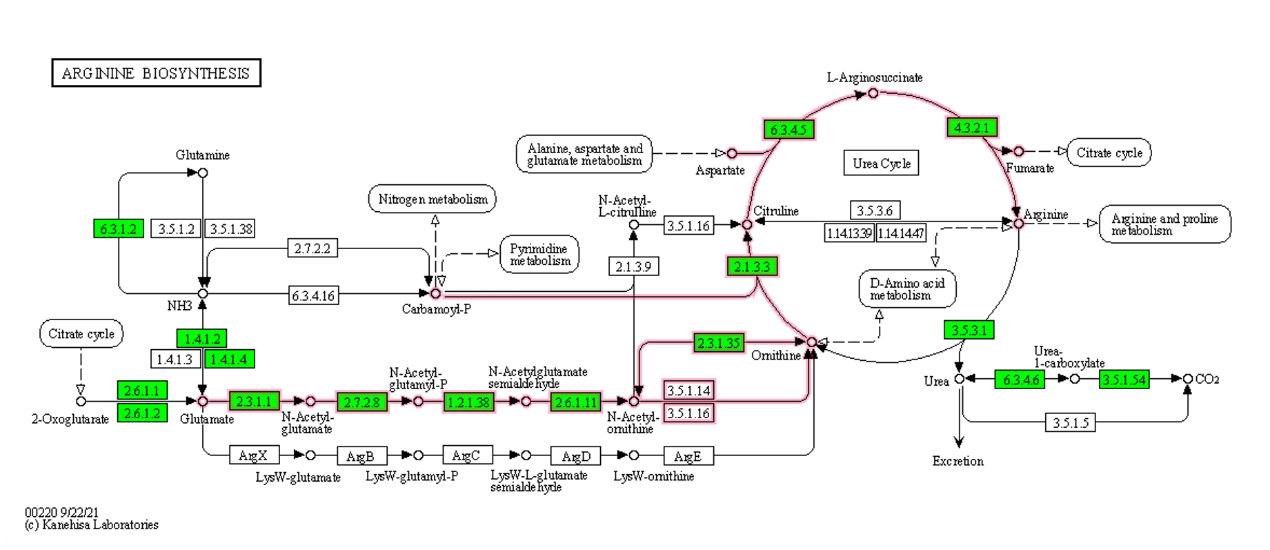


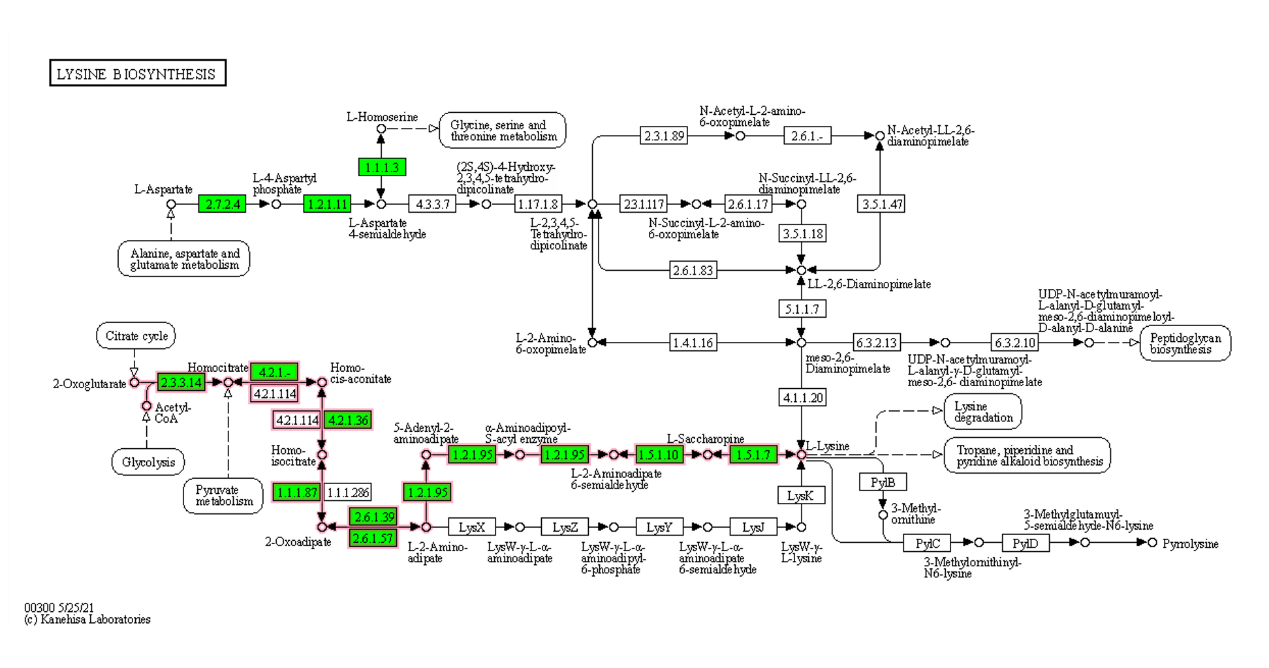


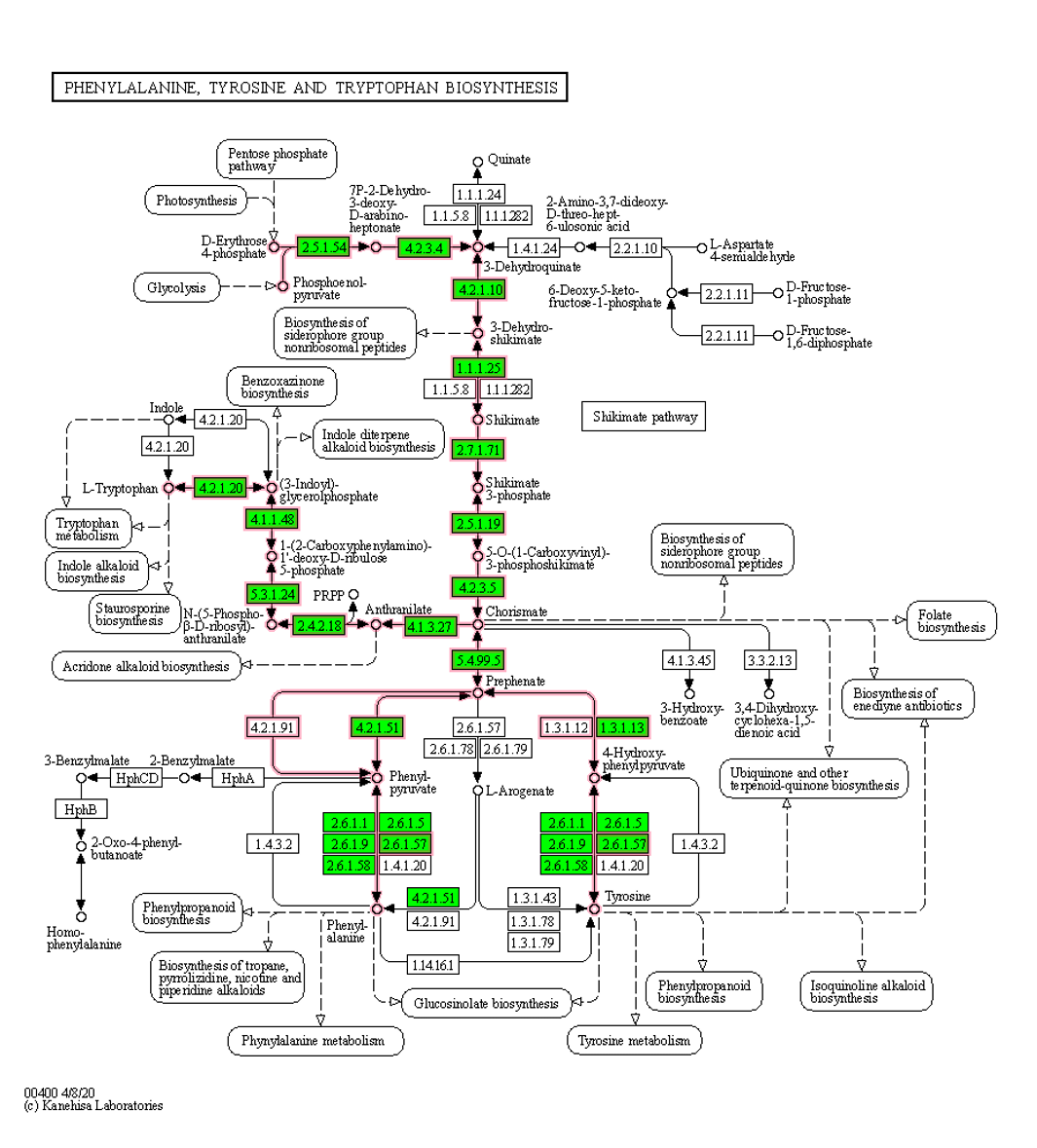


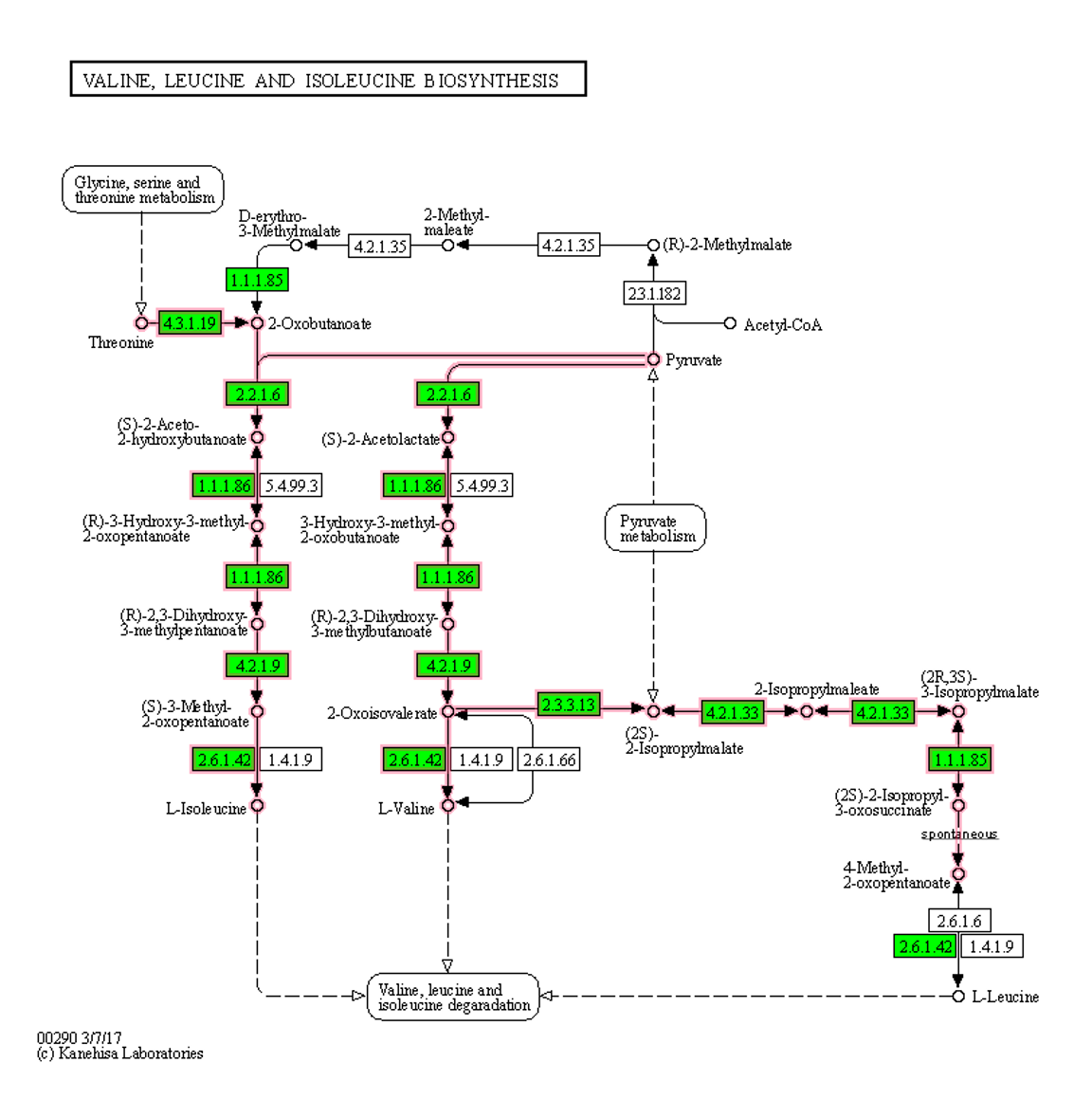


**Supplementary Figure 3**. Starch and sucrose metabolism in yeasts. Green boxes indicate the presence of the genes in the yeasts’ genomes.


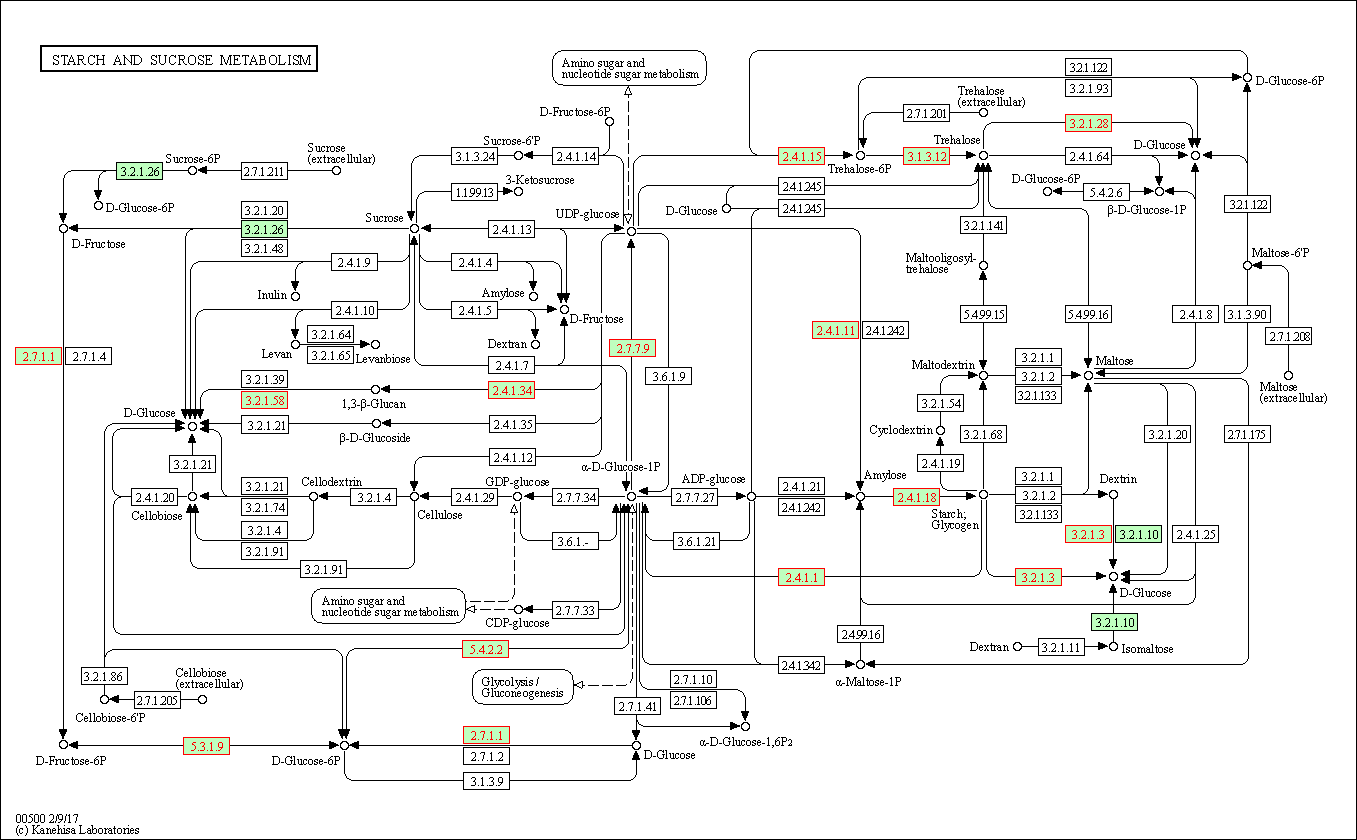


Starch and Sucrose metabolism in *Kuraishia molischiana*,


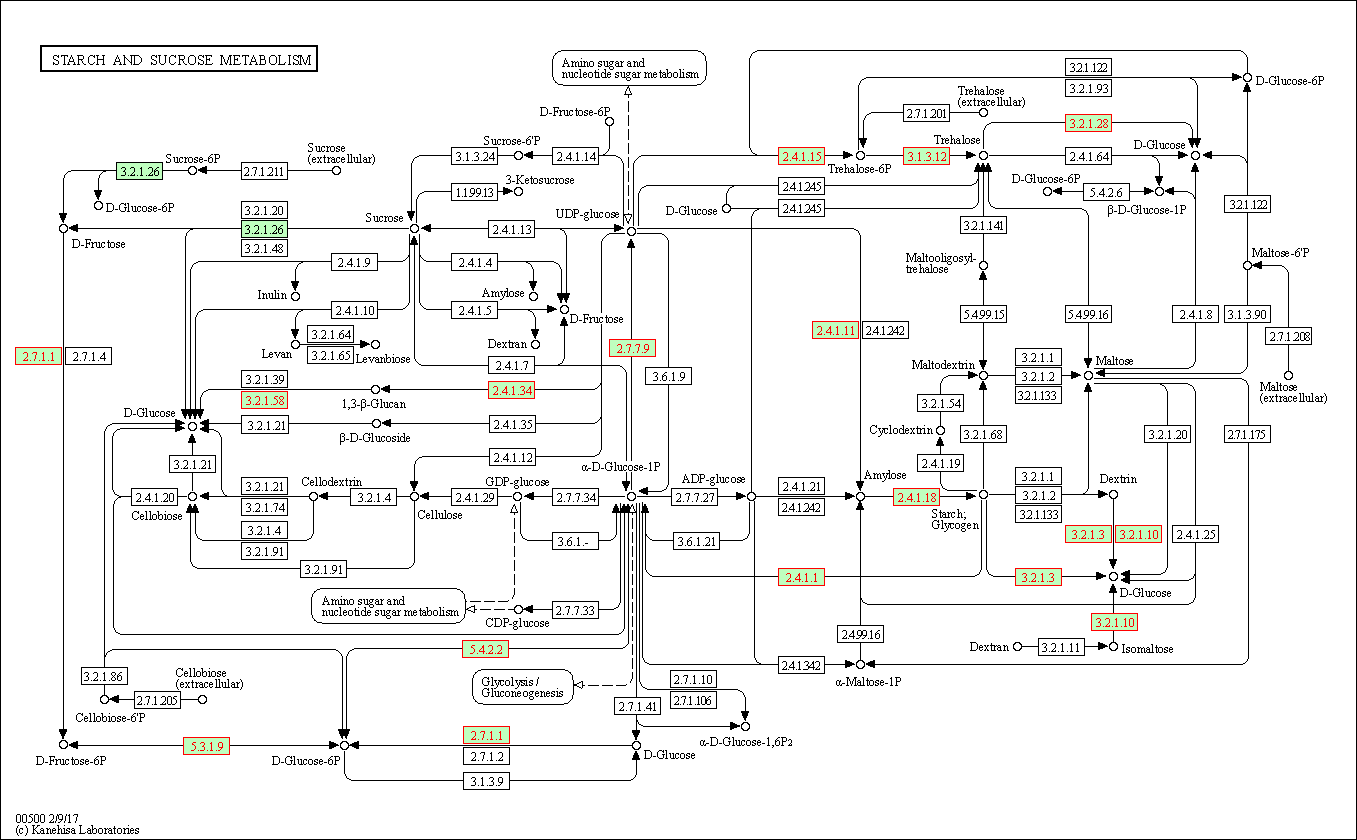


Starch and Sucrose metabolism in *Nakazawaea ambrosiae*


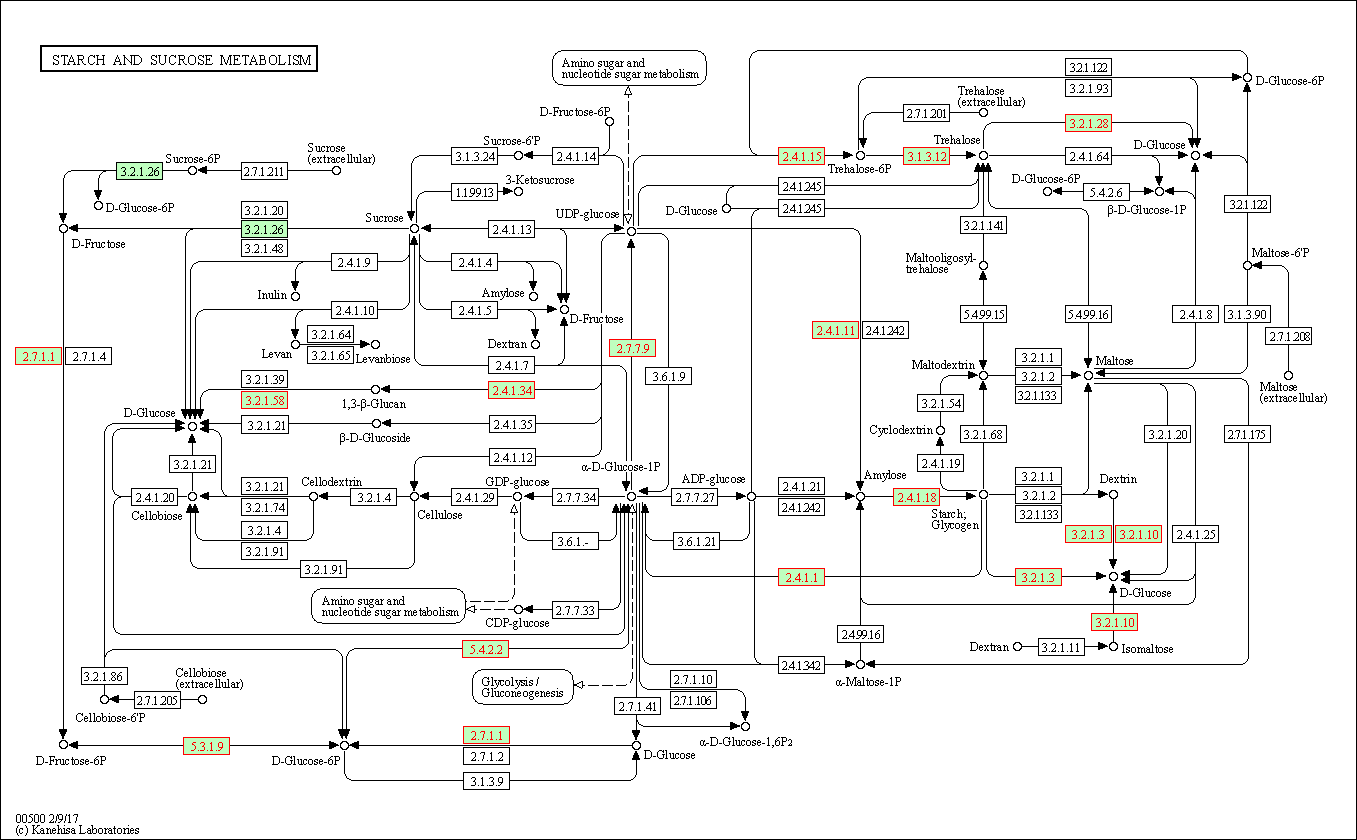


Starch and Sucrose metabolism in *Wickerhamomyces bisporus*


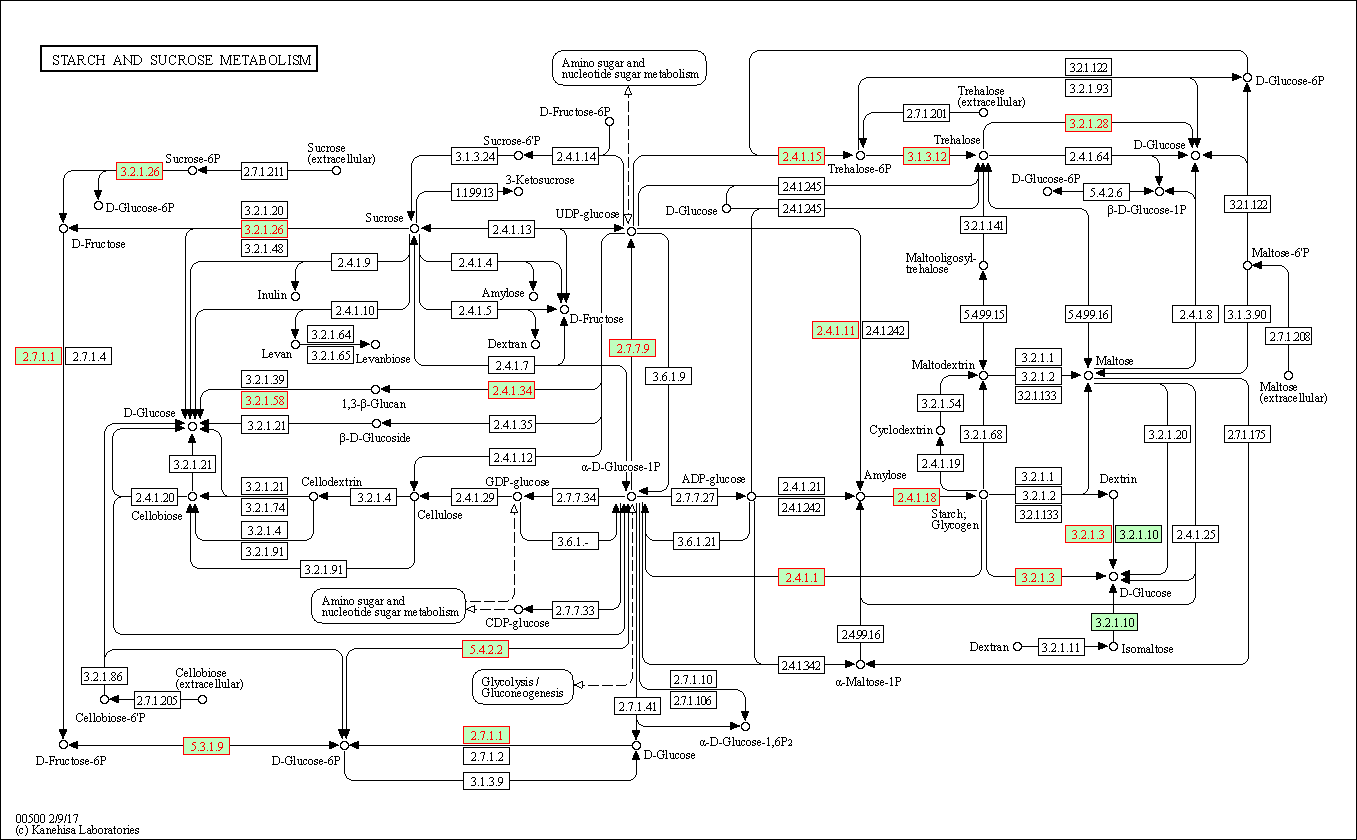


Starch and Sucrose metabolism in *Cryptococcus* sp


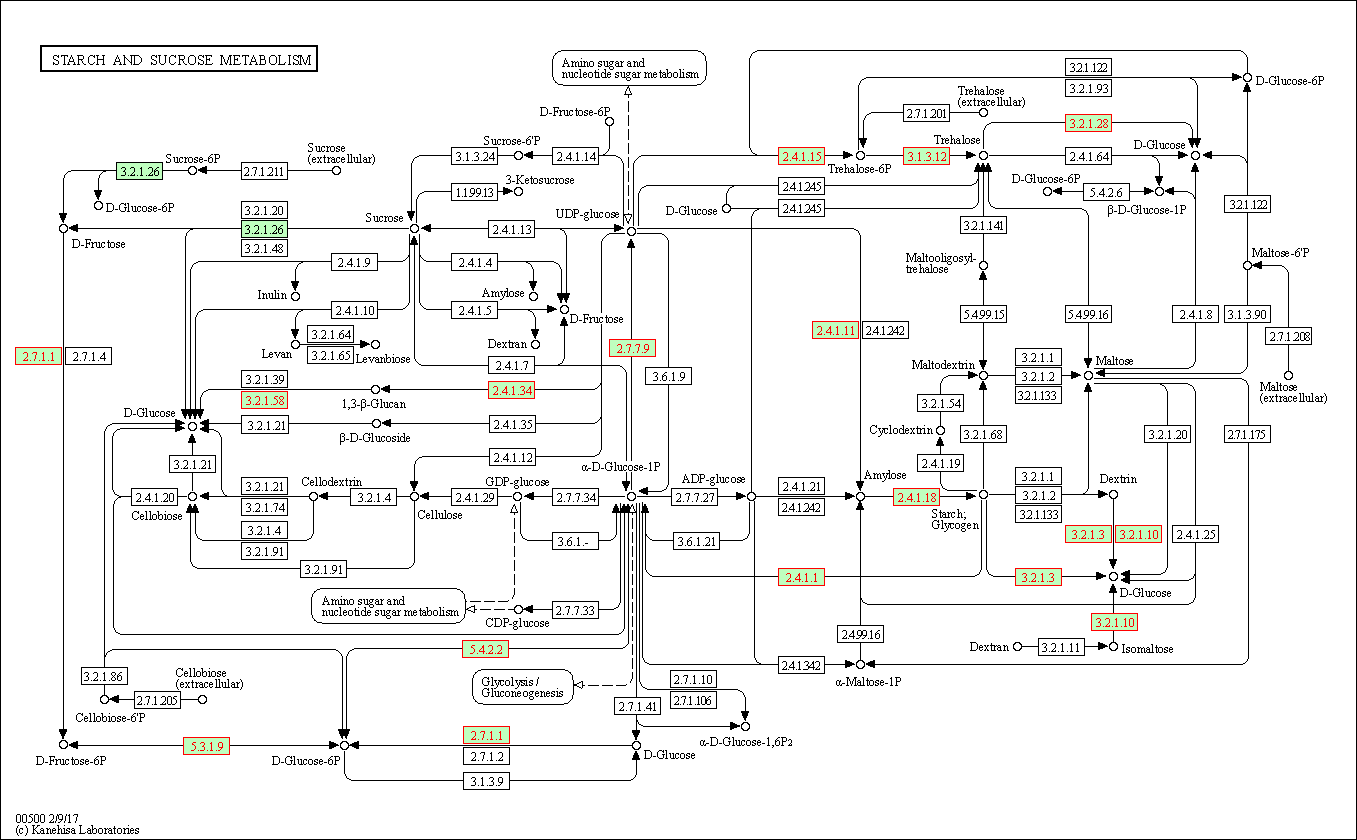


Starch and Sucrose metabolism in *Ogataea ramenticola*
